# Only One of Three Bcs1 Homologs in *Aspergillus fumigatus* Confers Respiratory Growth

**DOI:** 10.3390/jof9111074

**Published:** 2023-11-02

**Authors:** Isabel Klugherz, Marion Basch, Natanya Ng, Zhaojun Zhu, Nikola Wagener, Johannes Wagener

**Affiliations:** 1 Max von Pettenkofer-Institut für Hygiene und Medizinische Mikrobiologie, Medizinische Fakultät, Ludwig-Maximilians-Universität München, 80336 Munich, Germany; isabel.klugherz@medunigraz.at (I.K.);; 2 Diagnostic and Research Institute of Hygiene, Microbiology and Environmental Medicine, Medical University of Graz, 8010 Graz, Austria; 3 Zell- und Entwicklungsbiologie, Department Biologie II, Ludwig-Maximilians-Universität München, Planegg-Martinsried, 82152 Munich, Germany; 4 Department of Clinical Microbiology, School of Medicine, Trinity College Dublin, The University of Dublin, St James’s Hospital Campus, D08 RX0X Dublin, Ireland

**Keywords:** Bcs1, mitochondria, complex III, azoles, *Aspergillus fumigatus*

## Abstract

The mitochondrial translocase Bcs1 is required for the correct assembly of complex III of the mitochondrial respiratory chain. Because of its importance, Bcs1 was recently proposed as a target for antifungal agents. The function of this AAA (ATPase Associated with diverse cellular Activities) protein has been extensively characterized in *Saccharomyces cerevisiae*. This yeast as well as previously studied mammals each encode only one homolog. In contrast, the pathogenic mold *Aspergillus fumigatus* encodes three putative Bcs1 homologs, none of which have been characterized to date. To study the role of these three homologs in *A. fumigatus*, conditional and deletion mutants of the respective genes AFUA_3G13000 (*bcs1A*), AFUA_4G01260 (*bcs1B*), and AFUA_2G14760 (*bcs1C*) were generated. A deletion or downregulation of *bcs1A* resulted in drastically reduced growth and sporulation rates and in a significantly altered susceptibility to azole antifungals. In contrast, mutants lacking Bcs1B or Bcs1C did not show any phenotypes differing from the wild type. Salicylhydroxamic acid—an inhibitor of the alternative oxidase that allows the respiratory chain to bypass complex III in some species—caused a complete growth arrest of the *bcs1A* deletion mutant. In a *Galleria mellonella* infection model, the deletion of *bcs1A* resulted in significantly decreased virulence. Only Bcs1A was able to partially complement a deletion of *BCS1* in *S. cerevisiae*. The subcellular localization of Bcs1B and Bcs1C outside of mitochondria suggests that these Bcs1 homologs exert cellular functions different from that of Bcs1. Our data demonstrate that Bcs1A is the sole Bcs1 ortholog in *A. fumigatus*.

## 1. Introduction

The mitochondrial respiratory chain is one of the main sources of adenosine triphosphate (ATP) for most eukaryotic organisms. The respiratory chain of the well characterized baker’s yeast, *Saccharomyces cerevisiae*, includes two complexes that pump protons from the mitochondrial matrix into the intra-cristae lumen [1]. This leads to a proton gradient across the inner mitochondrial membrane which drives the ATP synthase-dependent ATP formation in the mitochondrial matrix. One of the two complexes is the cytochrome *bc*_1_ complex, which is also known as complex III, and which pumps protons based on the transfer of electrons from reduced ubiquinol to cytochrome *c*. The highly conserved Rieske iron–sulfur protein, in *S. cerevisiae* named Rip1, is an essential subunit for the electron transfer in the cytochrome *bc*_1_ complex. Biogenesis of this mitochondrial inner membrane protein is complex and requires several steps [2]. Rip1 is encoded in the nucleus, translated in the cytoplasm, and has to be imported across the two mitochondrial membranes into the mitochondrial matrix. There it is processed by the matrix processing peptidase and the mitochondrial intermediate peptidase, and inserted into the inner mitochondrial membrane. This circuitous route is required to equip Rip1 with an iron–sulfur cluster, specifically an 2Fe-2S cluster, which is inserted into the protein in the matrix—its place of origin [3,4]. The Rip1 iron–sulfur cluster binding domain is finally facing the intermembrane space and therefore translocated across and the transmembrane domain integrated into the mitochondrial inner membrane and assembled into the *bc*_1_ complex where it adopts a stably folded conformation [2,5].

Key for this final translocation step of Rip1 into the inner membrane is the highly conserved AAA (ATPase Associated with diverse cellular Activities) protein Bcs1 which acts as a translocase [4]. Mutations in the human *BCS1L* gene, which encodes a Bcs1 homolog, are associated with several human diseases such as encephalopathy, tubulopathy and hepatopathy, growth retardation, GRACILE (growth retardation, aminoaciduria, cholestasis, iron overload, lactacidosis, and early death) syndrome and Björnstad syndrome [6,7,8]. The yeast Bcs1 was shown to form homo-oligomeric complexes [9,10]. It shows an unexpected recently discovered property which distinguishes it from most other AAA proteins: it forms homo-heptameric instead of hexameric complexes [9,10]. It was hypothesized that this is a requirement to allow passage of the fully folded Rip1 protein including an iron–sulfur cluster. In human cells, a full-length BCS1L and possibly some shorter isoforms based on alternative splicing are expressed [11].

Bcs1 has been suggested as a potential target for new antifungals [12]. The mold *Aspergillus fumigatus*, a major fungal pathogen that causes life-threatening infections in immunocompromised patients [13], encodes three potential Bcs1 homologs. Here we show that only one of these three Bcs1 homologs in *A. fumigatus*, Bcs1A, is a functional orthologue of *S. cerevisiae* Bcs1. In agreement with an essential role in assembly of the cytochrome *bc*_1_ complex, a conditional *bcs1A* mutant phenocopied the characteristic growth defects of other complex III mutants under repressed conditions. This included the increased tolerance to azole antifungals. In agreement with the severe growth defect of mutants that lack Bcs1A, a Δ*bcs1A* deletion mutant showed a significantly reduced virulence in a *Galleria mellonella* infection model. Mutants that lack the two other Bcs1 homologs, Bcs1B or Bcs1C, had no apparent growth defects under the tested conditions and were as virulent as the wild type in the *G. mellonella* infection model. Further, the subcellular localizations of Bcs1B and Bcs1C were distinct from that of mitochondria, Bcs1C being localized in the nucleus in a cell cycle-dependent manner.

## 2. Material and Methods

### 2.1. Strains, Culture Conditions, and Chemicals

A non-homologous end joining-deficient *A. fumigatus* strain (AfS35, a derivative of D141 [14,15] served as the *A. fumigatus* wild-type strain in this study. Conditional mutants and deletion mutants of the genes encoding the putative Bcs1 homologs (*bcs1A*, AFUA_3g13000; *bcs1B*, AFUA_4g01260; *bcs1C*, AFUA_2g14760) were constructed by replacing the respective promoters with a doxycycline-inducible promoter (pkiA-tetOn; obtained from pYZ002 [16] or the genes with a self-excising hygromycin B resistance cassette (obtained from pSK528 [17], essentially as described before [18]. To visualize mitochondria with a mitochondria-targeted red fluorescent protein (mtRFP), strains were transformed with pYZ012. pYZ012 was constructed by replacing the GFP-encoding sequence in pCH005 [16] with the sequence encoding the monomeric red fluorescent protein 1 (mRFP1) obtained from pJW101 after digesting both plasmids with NotI and re-ligating the respective fragments [15]. Strains expressing C-terminally GFP-tagged Bcs1B or Bcs1C were constructed essentially as described previously [19]. Briefly, the coding regions without stop codons of *bcs1B* and *bcs1C* were amplified by PCR and cloned into the PmeI site of pJW103. The resulting constructs pIK002 and pIK003 were transformed into wild type. Experiments with *Aspergillus* were all repeated with at least two different clones of the same strain and performed on *Aspergillus* Minimal Medium (AMM; [20]) or Sabouraud medium (4% (*w/v*) d-glucose, 1% (*w/v*) peptone (LP0034; Thermo Fisher Scientific; Rockford, IL, USA, pH 7.0). For agar plates, 2% (*w/v*) agar (214030; Becton Dickinson; Franklin Lakes, NJ, USA) was added. The depicted experimental results are representative of at least three experiments performed under similar conditions, with the exception of the posaconazole Etest experiment, which was performed only twice, and the *Galleria mellonella* infection experiment, which was performed once as described below. All strains were maintained on Sabouraud medium to harvest conidia and incubated at 37 °C if not stated differently. All *A. fumigatus* strains used in this study are listed in Table 1.

All yeast strains were isogenic to W303 MATa wild type (W303a). The Δ*bcs1* deletion mutant was obtained from Nobrega et al. [21]. For the complementation experiment of this mutant, the strains were transformed with the plasmid pYX-242 (Novagen, Madison, WI, USA) which either harbored no additional sequence (controls; W303a and Δ*bcs1*), the sequence of yeast *BCS1* or the sequences of *bcs1A*, *bcs1B*, or *bcs1C* without the introns (cDNA), respectively. cDNA of *bcs1A*, *bcs1B* and *bcs1C* was obtained using the Thermo Scientific First Strand cDNA Synthesis Kit (#K1612; Thermo Scientific, Rockford, IL, USA) and RNA that was isolated from wild type, essentially as described before [22], using the QIAGEN RNeasy Mini Kit (#142349288; QIAGEN GmbH, Hilden, Germany). Yeast strains were cultured in SCGal medium (selective minimal medium supplemented with an amino acid mix but without leucine (SC), and with 2% (*w/v*) galactose (Gal)) and incubated at 30 °C. The logarithmic culture was harvested and washed, and the cell density was adjusted to an optical density (at 600 nm) of 1. A 1:10 dilution series was prepared in SCD (SC with 2% (*w/v*) glucose) or SCG (SC with 2% (*w/v*) galactose). Plates were subsequently incubated at 30 °C. Growth was documented using the ChemiDoc XRS+TM and Software Image LabTM 5.2.1 (Bio-Rad Laboratories, Hercules, CA, USA).

Calcofluor white (F3543), Salicylhydroxamic Acid (SHAM) (S607), and Congo red (60910) were obtained from Sigma-Aldrich (St. Louis, MO, USA). Doxycycline was obtained from Clontech (631311; Mountain View, CA, USA). Etest strips were obtained from bioMérieux (Marcyl’Etoile, France). Voriconazole was obtained from Apexbt Technology LLC (A4320; Houston, TX, USA). Mounting medium with DAPI was obtained from Abcam (ab104139, Cambridge, UK).

**Table 1:** *A. fumigatus* strains used in this work.

Strain or Genotype	Relevant Genetic Modification	Parental Strain	Reference
AfS35 (wt)	*akuA*::*loxP*	D141	[14]
Δ*bcs1A*	*bcs1A*::*loxP-hygro^R^*/*blaster*	AfS35 (wt)	This work
*bcs1 tetOn*	(*p*)*bcs1A*::*ptrA-*(*p*)*pkiA-tetOn*	AfS35 (wt)	This work
Δ*bcs1B*	*bcs1B*::*loxP-hygro^R^*/*blaster*	AfS35 (wt)	This work
*bcs1B_tetOn_*	(*p*)*bcs1B*::*ptrA-*(*p*)*pkiA-tetOn*	AfS35 (wt)	This work
Δ*bcs1C*	*bcs1C*::*loxP-hygro^R^/blaster*	AfS35 (wt)	This work
*bcs1C_tetOn_*	*(p)bcs1C*::*ptrA-*(*p*)*pkiA-tetOn*	AfS35 (wt)	This work
*Rip1* *_tetOn_*	*(p)Rip1*::*ptrA-*(*p*)*pkiA-tetOn*	AfS35 (wt)	[23]
wt + Bcs1B-GFP	pIK002	AfS35 (wt)	This work
wt + Bcs1C-GFP	pIK003	AfS35 (wt)	This work
wt + mtRFP	pYZ012	AfS35 (wt)	This work
wt + mtRFP + Bcs1B-GFP	pIK002	wt + mtRFP	This work
wt + mtRFP + Bcs1C-GFP	pIK003	wt + mtRFP	This work

### 2.2. Microscopy

Fluorescence microscopy was performed with a Leica SP5 confocal laser scanning microscope (Leica Microsystems, Mannheim, Germany). To visualize nuclei, conidia of the indicated strains were inoculated in Sabouraud medium on coverslips and cultured at 37 °C. After 10 h incubation, hyphae were fixed with 3.7% (*v/v*) formaldehyde for 3 min, washed with ddH_2_O and stained with 4′,6-diamidino-2-phenylindole (DAPI mounting medium). Live cell microscopy of strains expressing mitochondria-targeted red fluorescent protein was performed after inoculating conidia of the indicated strains in Sabouraud medium in 15 μ-Slide eight-well (#80826) slides (Ibidi; Martinsried, Germany) and incubating the slides for 10 h at 37 °C.

### 2.3. Galleria mellonella Infection Experiments

Virulence was analyzed in a *Galleria mellonella* infection model, essentially as described before [16]. Fresh larvae of the weight class 0.35 g to 0.55 g were obtained from AKM—Angel- und Ködermarkt, Munich, Germany. The larvae were divided in groups of 50 for each condition. Then, 5 × 10^5^ conidia in 10 µL ddH_2_O were injected per larva of the respective groups. The respective control groups were either injected with 10 µL ddH_2_O or not injected at all. The larvae were then incubated in the dark at 37 °C. Viability of the larvae was assessed every 8 h. Survival was plotted in a Kaplan–Meier curve in GraphPad Prism 5 for Windows, GraphPad Software, Boston, Massachusetts USA. Significance was calculated with IBM SPSS Statistics for Windows Version 27.0., IBM Corp., Armonk, NY, USA, using a log-rank (Mantel-Cox) test.

### 2.4. Bioinformatics and Databases

DNA and protein sequences were obtained from *Aspergillus* Genome Database (Af293) [24], FungiDB [25], the Saccharomyces Genome Database (SGD) [26], FlyBase [27], and National Center for Biotechnology Information (NCBI) [28]. Database searches were performed with the BLASTP tool of FungiDB (*C. albicans, S. pombe, A. fumigatus, A. nidulans, A. niger, A. flavus, F. oxysporum, M. circinelloides, N. crassa*), NCBI (*Homo sapiens, Mus musculus*) and FlyBase (*D. melanogaster*). Default settings were applied. For FungiDB, the cut-off for the e-value was set to 1 × 10^−20^. A reverse BLASTP search was performed for all results using SGD to confirm that Bcs1 is the top result for all included sequences. Jalview [29] and MEGA11 [30] were used to perform alignments and to visualize the relatedness of sequences.

## 3. Results

### 3.1. A. fumigatus Encodes Three Bcs1 Homologs

**Figure 1:**
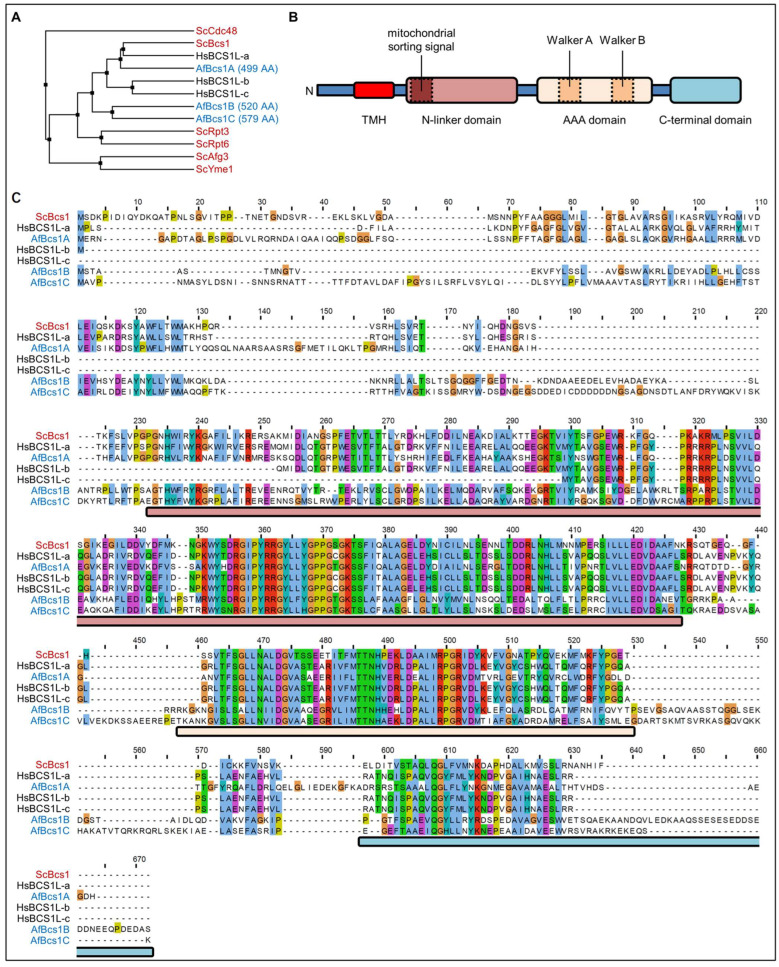
*A. fumigatus* encodes three homologs of *S. cerevisiae* Bcs1 and *H. sapiens* BCS1L. (**A**) Average distance tree and alignment of the protein sequences of *S. cerevisiae* Bcs1 (ScBcs1), the three *H. sapiens* BCS1L isoforms (HsBCS1L-a, -b, and -c), and the three ScBcs1/HsBCS1L homologs encoded in the genome of *A. fumigatus* (AfBcs1A, AfBcs1B, AfBcs1C) as well as of closely related *S. cerevisiae* AAA proteins (ScCdc48, ScRpt3, ScRpt6, ScAfg3, and ScYme1). Amino acid (AA) lengths of *A. fumigatus* homologs are annotated next to the respective protein names. The average distance tree was generated with BLOSUM62. (**B**) Schematic representation of the Bcs1 structure featuring a transmembrane domain near the N terminus, a mitochondrial sorting signal in the N-linker domain, the conserved Walker A and Walker B motifs in the AAA domain, plus the so far uncharacterized C-terminal domain. (**C**) Alignment (T-Coffee; Clustal color scheme) of the protein sequences of *S. cerevisiae*, *H. sapiens*, and *A. fumigatus* Bcs1 homologs. The approximate regions encompassing the domains indicated in (**B**) are highlighted in the respective colors.

We recently showed that *A. fumigatus* mutants that lack a functional cytochrome *bc*_1_ complex exhibit a reduced susceptibility to the fungicidal effect of azole antifungals [23]. Baker’s yeast and *Homo sapiens* as well as other explored metazoa and fungi, i.e., *Mus musculus*, *Drosophila melanogaster*, *Candida albicans*, and *Schizosaccharomyces pombe*, each harbor only one unambiguous gene that encodes the obvious Bcs1 homolog. During our investigations, we unexpectedly found three genes in the genome of *A. fumigatus*, AFUA_3G13000, AFUA_4G01260, and AFUA_2G14760, which encode putative Bcs1 homologs. The genes were named *bcs1A*, *bcs1B*, and *bcs1C*, respectively (Figure 1).

To initially appraise the possible roles of the three *A. fumigatus* Bcs1 homologues, we analyzed the protein sequences of Bcs1 from baker’s yeast, the different human Bcs1 transcriptional variants and the three homologues from *Aspergillus* by alignment. The more extensively studied Bcs1 homologs in human and baker’s yeast share certain features (Figure 1B). As shown in Figure 1C, most of these features were also found in the three Bcs1 homologs encoded in the genome of *A. fumigatus*. Bcs1 functions in a homo-oligomeric complex as a translocase for the Rieske protein in the inner mitochondrial membrane [4]. For this purpose, it contains a transmembrane domain which is located close to its N terminus that is required for mitochondrial import [21,31]. Our alignment revealed that Bcs1A and Bcs1C both harbor such a putative N-terminal transmembrane domain, while it appeared to be lacking in Bcs1B (Figure 1C). N-terminal to the Bcs1 transmembrane segment found in *S. cerevisiae* and *H. sapiens* is a short soluble stretch whose function is currently unknown. The alignment demonstrated that Bcs1A and Bcs1C of *A. fumigatus* also harbor such a stretch (Figure 1C). Interestingly, Bcs1 uses a very unusual signal sequence for its import via the TOM and TIM23 translocase to its destination in the inner mitochondrial membrane: it forms the required amphipathic helix structure by building a loop of the transmembrane domain and the following positively charged stretch consisting of amino acid residues 69 to 126 [2,31], which seems to be less conserved in both Bcs1B and Bcs1C. Like all AAA proteins, Bcs1 contains a conserved Walker A motif which is required for nucleotide binding. Downstream of the Walker A motif, a conserved Walker B motif can be found, which in turn is required for the hydrolysis of the bound nucleotide. The Walker B motif contains an essential acidic amino acid residue that, if mutated into a basic residue, renders the AAA protein dysfunctional. Both motifs are located within the canonical AAA domain, which is usually highly conserved between homologs in different species, but even between functionally unrelated proteins [32]. A Bcs1-specific N-linker domain is found between the transmembrane domain and the AAA domain. As shown in Figure 1C, this is also conserved among all Bcs1 proteins, including the *A. fumigatus* Bcs1A, Bcs1B, and Bcs1C, but not found in other proteins of the AAA family. Interestingly, more severe phenotypes in patients with BCS1L mutations are often linked to mutations in this Bcs1-specific domain [33,34]. Structural analyses of Bcs1 suggest that this domain forms a loop protruding into the middle of the oligomeric complex to form the seal required to prevent the formation of a hole in the membrane. In addition, it provides a contact point between neighboring subunits of the Bcs1 heptamer [9]. Downstream of the AAA domain, Bcs1 proteins contain a partially conserved C-terminal domain, the function of which is unknown so far. Our analysis also revealed such a domain in the three *A. fumigatus* Bcs1 homologs (Figure 1C). Furthermore, Bcs1B and Bcs1C showed C-terminal extensions upstream and downstream of this domain that are highly conserved among all Bcs1 proteins (Figure 1C).

### 3.2. Construction and Phenotypic Characterization of bcs1A, bcs1B, and bcs1C Mutants

**Figure 2:**
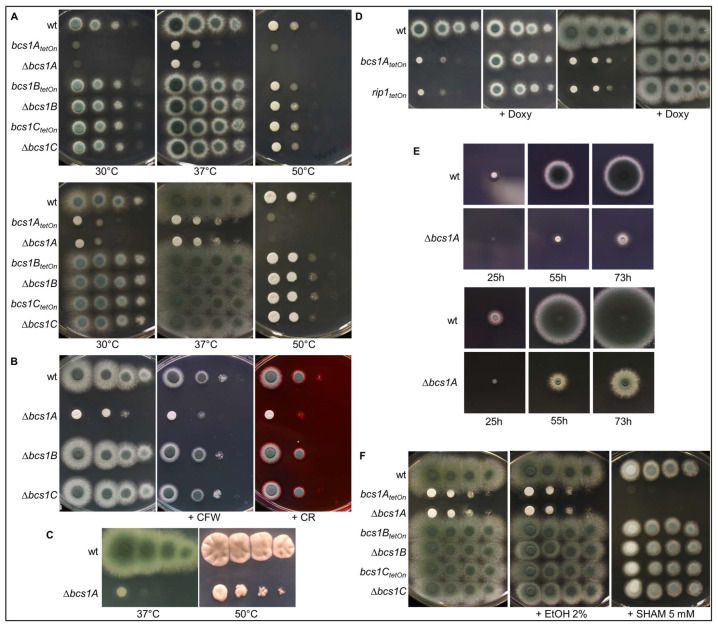
Only deletion or conditional downregulation of *bcs1A*, but not of *bcs1B* or *bcs1C*, results in impaired growth of *A. fumigatus*. (**A**–**D**,**F**) In a series of 10-fold dilutions derived from a starting suspension of 5 × 10^7^ conidia mL^−1^ of the indicated strains, aliquots of 3 μL were spotted onto an AMM (**A**, upper panel; **D** left panel) or Sabouraud (**A**, lower panel; **B**; **C**; **D** right panel; **F**) agar plate. When indicated, plates were supplemented with 50 μg/mL calcofluor white (+CFW), 70 μg/mL Congo red (+CR), 15 μg/mL doxycycline (+Doxy), 2% (*v/v*) ethanol (+EtOH, control), or 5 mM salicylhydroxamic acid in EtOH (+SHAM; final ethanol concentration 2% (*v/v*)). (**E**) From a conidia suspension of 5 × 10^7^ conidia ml^−1^ of the indicated strains, 3 μL was spotted in the center of AMM (upper panel) or Sabouraud (lower panel) agar plates. All plates were incubated at 37 °C if not labeled differently for the following time periods: (**A**,**D**,**F**) 36 h; (**B**) 28 h; (**C**) left image 72 h, right image 120 h; (**E**) for the indicated incubation times.

To investigate the roles of the three different *Aspergillus* Bcs1 homologs, deletion and conditional mutants of the respective genes were constructed. To construct the deletion mutants, a hygromycin resistance cassette was used to replace the coding regions of the genes [17]. To construct the conditional mutants a doxycycline-inducible Tet-On promoter was used to functionally replace the genes’ native promoters [18]. Only the *bcs1A* mutants exhibited growth phenotypes that differed from the wild type. Neither the Δ*bcs1B* deletion mutant nor the conditional *bcs1B_tetOn_* mutant under repressed conditions showed growth defects on complex or minimal media or when exposed to stress conditions, i.e., increased temperature or cell wall perturbing agents such as Congo red and calcofluor white (Figure 2A,B). The same applies to the Δ*bcs1C* deletion mutant and the conditional *bcs1C_tetOn_* mutant (Figure 2A,B). In contrast, as shown in Figure 2A, growth of the Δ*bcs1A* deletion mutant as well as of the conditional *bcs1A_tetOn_* mutant under repressed conditions was drastically impaired under normal growth conditions. The growth resembled that of a conditional *rip1_teton_* mutant when it was cultured under similar conditions (Figure 2D and Supplementary Figure S1). While deletion of *bcs1A* resulted in a significantly delayed radial growth, the Δ*bcs1A* mutant retained its ability to form conidia after extended growth periods (Figure 2E). Induction of the conditional promoters with doxycycline fully recovered growth of the conditional *bcs1A_tetOn_* and *rip1_teton_* mutants (Figure 2D and Supplementary Figure S1). Interestingly, loss of Bcs1A did not significantly alter the resistance of *A. fumigatus* to the cell wall perturbing agents calcofluor white and Congo red nor resulted in an increased susceptibility to heat (Figure 2B,C).

In contrast to baker’s yeast, *A. fumigatus* expresses an alternative oxidase that uses ubiquinol as a substrate and thus enables the mold to bypass complex III [35]. As shown in Figure 2F, the specific alternative oxidase inhibitor salicylhydroxamic acid (SHAM) fully suppressed growth of the Δ*bcs1A* mutant and the conditional *bcs1A_tetOn_* mutant under repressed conditions. This shows that Bcs1A is essentially required for the conventional mitochondrial electron transport chain and that Bcs1B and Bcs1C cannot compensate for the loss of Bcs1A.

### 3.3. Expression of A. fumigatus bcs1A Partially Complements the BCS1Deletion in S. cerevisiae

**Figure 3:**
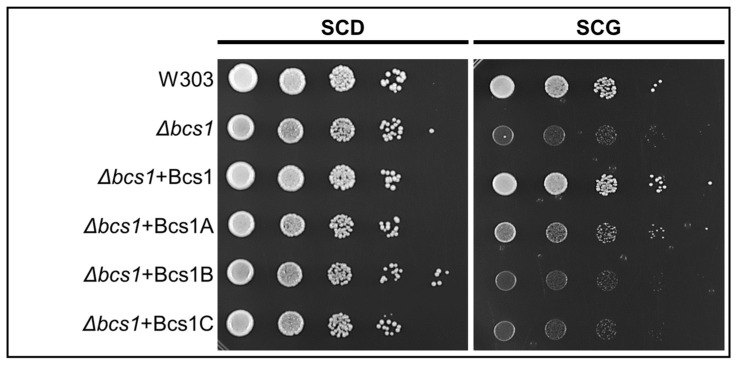
*A. fumigatus* Bcs1A can partially complement the lack of Bcs1 in *S. cerevisiae*. In series of 10-fold dilutions derived from starting suspensions with optical densities (at 600 nm) of 1 of the indicated strains, aliquots of 3.5 μL were spotted on a SCD or SCG agar plate and incubated for 48 h at 30 °C. Compared to the *S. cerevisiae* wild type (W303a), the Δ*bcs1* deletion mutant showed a growth defect on SCG medium which was complemented by yeast Bcs1 and partially complemented by *A. fumigatus* Bcs1A. *A. fumigatus* Bcs1B and Bcs1C did not complement the growth defect.

Our data suggested that Bcs1A but not Bcs1B and Bcs1C is the only functional orthologue of *S. cerevisiae* Bcs1 in *A. fumigatus*. We asked whether any of the Bcs1 homologs of *A. fumigatus* can functionally complement the lack of Bcs1 in *S. cerevisiae*. To this end, we transformed a Δ*bcs1* yeast strain with constructs that express either the coding regions of the *A. fumigatus* Bcs1 homologs or the coding region of yeast Bcs1 as a control. As shown in Figure 3, deletion of *BCS1* resulted in impaired growth of *S. cerevisiae* on medium with a non-fermentable carbon source. Reintroduction of *BCS1* into this strain reconstituted growth to a level similar to that of the wild type. The expression of Bcs1A partially complemented the growth defect of the Δ*bcs1* strain. In contrast, the transformation of the Δ*bcs1* strain with similar expression plasmids that harbor the coding sequences of Bcs1B or Bcs1C did not improve the growth on non-fermentable carbon source. These results support the conclusion that Bcs1A is the functional orthologue of the yeast Bcs1 and that the role of Bcs1 is essentially conserved between *S. cerevisiae* and *Aspergillus*.

### 3.4. Deletion of bcs1 Leads to an Increased Growth-Inhibiting but Decreased Fungicidal Activity of Azole Antifungals in A. fumigatus

We have previously shown that the lack of a functional conventional mitochondrial electron transport chain results in an altered susceptibility of the *A. fumigatus* to azole antifungals [23]. The reduced expression of the Rieske protein (Rip1) or of cytochrome *c* (CycA) led to a reduced minimal inhibitory concentration (MIC) of voriconazole and to a decrease in its remarkable fungicidal activity observed in *Aspergillus*. We therefore analyzed whether the deletion of any of the Bcs1 homologs-encoding genes in *A. fumigatus* changes the antifungal susceptibility.

**Figure 4:**
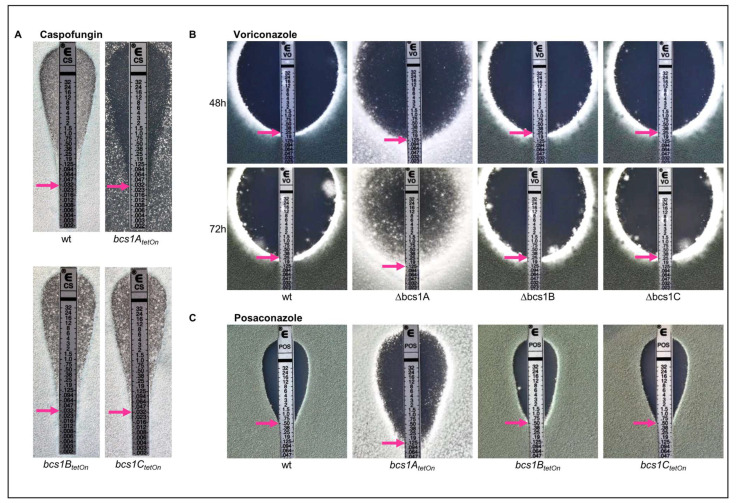
The absence of Bcs1A but not of Bcs1B or Bcs1C results in altered azole susceptibility. Sabouraud agar plates were inoculated with 1 × 10^6^ conidia of the indicated strains. Etest strips were applied and the plates were incubated at 37 °C. Representative photos of caspofungin were taken after 28 h (**A**), of voriconazole after 48 h and an extended period of 72 h (**B**) and of posaconazole after 48 h (**C**). Red arrows indicate the minimal inhibitory concentrations.

Bcs1A, Bcs1B, and Bcs1C appeared to be dispensable for the growth in the presence of echinocandin antifungals which target the fungal cell wall, since the wild type and the conditional mutants under repressed conditions showed similar caspofungin inhibition zones (Figure 4A). Deletion or conditional repression of *bcs1A*, but not of *bcs1B* or *bcs1C*, resulted in a slightly decreased MIC of azole antifungals such as voriconazole and posaconazole (Figure 4B,C). Notably, the decrease in the MIC of voriconazole observed with the conditional *bcs1A_tetOn_* mutant under repressed conditions was very similar to the decrease observed with the conditional *rip1_teton_* mutant under repressed conditions (Supplementary Figure S1). Interestingly, and very similarly to the previously described conditional *rip1_teton_* and *cycA_tetOn_* mutants [23], the lack of Bcs1A resulted in the ability of *A. fumigatus* to substantially grow within the inhibition zone of the azoles (Figure 4B,C), which was best visible after extended incubation (Figure 4B). In contrast, the inhibition zones of the azoles with wild type as well as with the mutants that lack Bcs1B or Bcs1C remained clear except for an expected number of naturally occurring azole-resistant clones which are based on spontaneous mutations (Figure 4B,C).

### 3.5. Subcellular Localizations of Bcs1B and Bcs1C Suggest Functional Roles Distinct from That of Bcs1A

**Figure 5:**
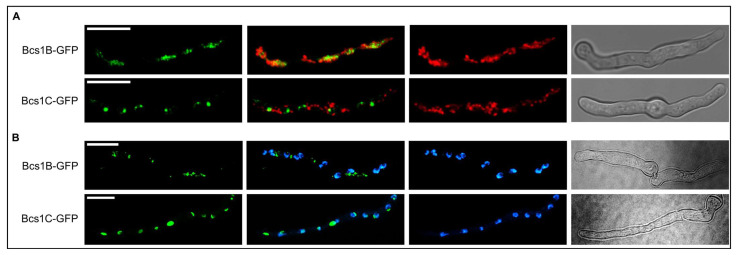
Bcs1B and Bcs1C show distinct localizations outside of mitochondria. (**A**) Conidia of wild type expressing C-terminally GFP-tagged Bcs1B (Bcs1B-GFP) or Bcs1C (Bcs1C-GFP) and mitochondria-targeted red fluorescent protein (RFP) were inoculated in Sabouraud medium and incubated at 37 °C for 10 h. Hyphae were then analyzed without fixation at 37 °C with a confocal laser scanning microscope. For each panel from left to right: GFP signal, overlay of GFP and RFP signal, RFP signal, bright field signal. (**B**) Conidia of wild type expressing C-terminally GFP-tagged Bcs1B (Bcs1B-GFP) or Bcs1C (Bcs1C-GFP) were inoculated in Sabouraud medium and incubated at 37 °C for 10 h. Hyphae were fixed in 3.7% (*v/v*) formaldehyde, stained with DAPI, and analyzed with a confocal laser scanning microscope. For each panel from left to right: GFP signal, overlay of GFP and DAPI signal, DAPI signal, bright field signal. (**A**,**B**) GFP, RFP, and DAPI images represent z-stack projections of the entire hyphae in focus, bright field images represent single focal planes. Bars represent 10 μm and are applicable to all images of the respective panel.

Bcs1A is important for complex III function and thus most likely has a similar role to its counterparts in baker’s yeast and human, the mitochondria-localized *S. cerevisiae* Bcs1 and *Homo sapiens* BCS1L [21,36,37]. However, our results indicated that Bcs1B and Bcs1C are not functional Bcs1 orthologs. We questioned whether these proteins are actually mitochondrial proteins. To visualize the subcellular localization of Bcs1B and Bcs1C in *A. fumigatus*, we expressed C-terminally green fluorescent protein (GFP)-tagged versions of these proteins in the wild type and in a wild-type strain that express a mitochondria-targeted red fluorescent protein. Both, Bcs1B-GFP and Bcs1C-GFP showed localization patterns within the hyphae that were distinct from that of mitochondria (Figure 5A). Interestingly, Bcs1C-GFP, but not Bcs1B-GFP, showed a remarkable co-localization with 4′,6-diamidino-2-phenylindole (DAPI)-stained nuclei (Figure 5B). This suggests that both Bcs1B and Bcs1C exert cellular functions outside of mitochondria and that Bcs1C presumably has a nuclear function.

### 3.6. Bcs1A Is Required for Full Virulence of A. fumigatus in a Galleria mellonella Infection Model

**Figure 6:**
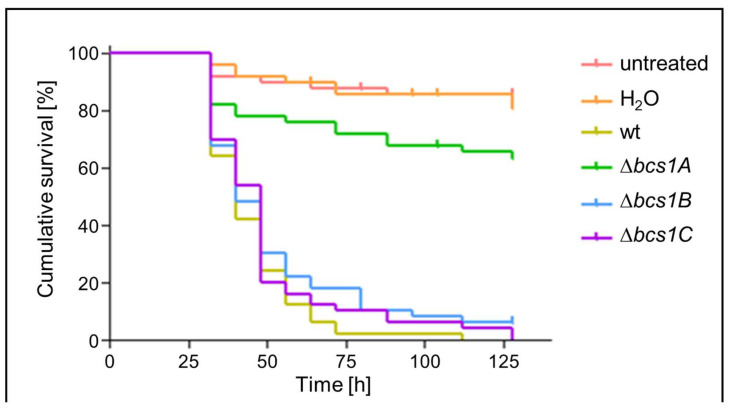
*Galleria mellonella* larvae infected with the Δ*bcs1A*-strain survive significantly longer. *G. mellonella* larvae were inoculated in groups of ten with 5 x 10^5^ conidia of the indicated strains, each resuspended in 10 µL of deionized water. The control groups were either inoculated with 10 µL of deionized water or left untreated. Larvae were maintained at 37 °C in the dark. Viability of the larvae was evaluated every 8 h for a total period of 128 h. The Kaplan–Meier graph shows the cumulative survival of all 50 larvae of each group. *p*-values: Δ*bcs1A* vs. wt: <0.001, Δ*bcs1B* vs. wt: 0.087; Δ*bcs1C* vs. wt: 0.226.

Finally, we assessed whether Bcs1A, Bcs1B, or Bcs1C is important for the virulence of *A. fumigatus* in a *Galleria mellonella* infection model. *Galleria* larvae were infected with conidia of the wild type or the Δ*bcs1A*, Δ*bcs1B*, or Δ*bcs1C* deletion mutants. As shown in Figure 6, the virulence of the Δ*bcs1B* and Δ*bcs1C* deletion mutants was similar to that of the wild type. In contrast, the virulence of the Δ*bcs1A* mutant was significantly reduced compared to the wild type (Figure 6). Notably, the virulence of the Δ*bcs1A* mutant appeared not fully attenuated when compared to the non-infected controls. This demonstrates that Bcs1A contributes to virulence of *A. fumigatus*, which is in line with its mitochondrial function in assembling complex III and a renowned role of mitochondrial respiration in fungal virulence [35,38].

## 4. Discussion

Interestingly, the genome of *A. fumigatus* encodes three Bcs1 homologs. This is in contrast to other species where the functional role of Bcs1 orthologs has been explored in detail. Our study demonstrates that only one of these three homologs, Bcs1A, is a functional ortholog of Bcs1. While mutants lacking Bcs1A show a severe growth defect, that is, drastically reduced radial growth as well as reduced formation of conidia, the deletion of *bcs1B* or *bcs1C* did not result in any apparent growth phenotypes. *A. fumigatus* essentially depends on a functional mitochondrial respiratory chain. The finding that the Δ*bcs1A* mutant is viable could therefore indicate that either one of the other Bcs1 homologs is partially functionally redundant with Bcs1A or, based on the literature, that the alternative oxidase can compensate for the loss of complex III [35]. Our results show that it is primarily the alternative oxidase that compensates for the loss of complex III functionality in the absence of Bcs1A since the alternative oxidase inhibitor SHAM fully suppressed growth of the Δ*bcs1A* mutant.

The expression of *A. fumigatus* Bcs1A in *S. cerevisiae* partially complemented the *BCS1* deletion. While it cannot be fully excluded that Bcs1A complements the *S. cerevisiae* Δ*bcs1* mutant by means other than direct complementation of the Bcs1 function as an insertase of Rip1 in complex III, this further supports the conclusion that Bcs1A is the functional Bcs1 ortholog in *A. fumigatus*. Interestingly, experiments where a Δ*bcs1* deletion mutant in *S. cerevisiae* was complemented by expressing BCS1L, the human Bcs1 homolog, also yielded a strain still exhibiting a severe growth phenotype, while the expression of the respective yeast protein fully rescued the Δ*bcs1* deletion mutant [39]. This implies functional and structural differences between Bcs1 proteins of different species.

What could be the function of the other two Bcs1 homologs which are not involved in the assembly of complex III? An analysis of the annotated genome sequences of selected animals and fungal species demonstrates that Bcs1B and Bcs1C homologs appear to be commonly found in molds, but not in animals and yeasts. Animals such as *H. sapiens*, *M. musculus*, and *D. melanogaster* and yeast such as *S. cerevisiae*, *C. albicans*, and *S. pombe* each encode only one Bcs1 homolog. In contrast, all molds we have analyzed appear to encode multiple Bcs1 homologs. For example, similarly to *A. fumigatus*, *Aspergillus niger* encodes two homologs, *Aspergillus nidulans* three homologs, and *Aspergillus flavus* five homologs. *Fusarium oxysporum*, *Mucor circinelloides*, and *Neurospora crassa* encode eight, five, or three homologs, respectively. Based on the homologies of the different Bcs1 homologs encoded in molds and yeasts (Supplementary Figure S2) we propose that only one of the multiple homologs per analyzed mold species represents a functional Bcs1 ortholog. The other Bcs1 homologs probably have similar functions as Bcs1B and Bcs1C in *A. fumigatus*. Our Δ*bcs1B* and Δ*bcs1C* mutants did not show any obvious growth phenotypes when compared to the wild type. We could therefore not assign a specific function to the respective genes. However, our analysis of the subcellular localization demonstrates that both proteins most likely exert different functions because they localize to different subcompartments of the fungal cell. Our analysis of the protein sequences of these homologs showed that in Bcs1B, no N-terminal transmembrane domain can be predicted which is an essential prerequisite for mitochondrial import of Bcs1 in yeast [31]. It was shown that in addition to this transmembrane domain, a subsequent positively charged stretch is required for the successful import of Bcs1. This stretch is less conserved in Bcs1B and Bcs1C, which could prevent the mitochondrial sorting for both Bcs1B and Bcs1C. Interestingly, both proteins contain amino acid stretches around the conserved C-terminal domain which are not found in yeast Bcs1, human BCS1L, or AfBcs1A. Perhaps these sections contain targeting signals for other subcompartments in the cell, for example, a nuclear localization signal in the case of Bcs1C. Although purely speculative, given the function of Bcs1 as an insertase in mitochondria, Bcs1C could also play a similar role in the insertion of proteins into the nuclear membrane. Understanding the function of Bcs1C might be an interesting future research question.

In agreement with our previous analysis of conditional *rip1* and *cycA* mutants, the *bcs1A* mutant shows an astonishing susceptibility to azole antifungals when compared to the wild type. Azoles normally have fungicidal activity in *A. fumigatus* [23]. Because of this, hyphae typically do not survive within the inhibition zone on solid media. However, the mutants lacking Bcs1A showed minimal growth in the azole inhibition zone as well as a significantly decreased MIC, very similarly to what we previously found for mutants lacking CycA or Rip1 [23]. This strongly suggests that this phenotype is linked with an overall dysfunction of the complex III. Interestingly, Vincent et al. found exactly the opposite effect for the pathogenic yeast *C. albicans* [40]. Azoles, which have only a fungistatic effect against yeasts, become fungicidal when combined with a *bc_1_*-inhibitor [40]. The molecular mechanisms behind this are puzzling and require further investigation.

The respiratory chain and its complex III are established targets for antiparasitic and antifungal agents [41]. Bcs1, which is essential for the assembly of complex III, was proposed as a novel target for antifungal therapy [12]. Our results demonstrate that the Bcs1 ortholog Bcs1A, in contrast to the other two Bcs1 homologs with distinct functions, contributes to the virulence of *A. fumigatus* in a *G. mellonella* infection model. This is in agreement with the reduced virulence of an *A. fumigatus* cytochrome *c* mutant in a murine infection model [35]. Notably, in both infection models, the virulence of the respective mutants was not completely abolished. The residual virulence still present in these models could be explained with the activity of the alternative oxidase and bypassing of complex III. In fact the alternative oxidase inhibitor SHAM fully suppresses growth in vitro of both the Δ*bcs1A* and Δ*cycA* mutants (this study, and [35]). Several important and clinically used antifungals do not have a pure fungicidal effect [42,43]. It is therefore well possible that inhibition of complex III alone has sufficient fungistatic potential to successfully treat or suppress infections in the human host. At least in agriculture, complex III inhibitors such as amisulbrom and ametoctradin are successfully used as fungicides, even though many phytopathogenic fungi express an alternative oxidase. But the expression of the alternative oxidase remains a concern in relation to possible resistance mechanisms against these drugs [41,44]. Many human pathogenic fungi harbor no alternative oxidase and therefore could not exploit this evasion strategy. In addition, co-treatment with specific inhibitors of the alternative oxidase, which is not found in mammalian cells, could surpass the limitations of complex III inhibition alone [41]. Should it be possible to generate specific inhibitors for fungal Bcs1 orthologs which do not interfere with human BCS1L, their use as a therapeutic agent for fungal infections should be considered.

## Data Availability

Data are available from the corresponding authors upon reasonable request.

## Supplementary Materials

The following supporting information can be downloaded at: https://www.mdpi.com/article/10.3390/jof9111074/s1, Figure S1. Reduced expression of *bcs1A* and *rip1* results in a comparable change in azole susceptibility. 1 × 10^6^ conidia of the indicated strains were spread on Sabouraud agar plates. Etest strips were applied, and representative images were taken after 48 h incubation at 37 °C. Figure S2. A neighbor-joining tree of the alignment of *S. cerevisiae*’s Bcs1 and its homologues within the indicated species was created using MEGA11. Firstly, the protein sequences were aligned using the ClustalW algorithm, then a neighbor-joining tree was generated. The values above the branches indicate the percentage of replicate trees in which the associated taxa clustered together in the bootstrap test (500 replicates). Homologues that are annotated as Bcs1 or putative Bcs1 display this information next to the respective identifier. All other homologs were annotated as hypothetical proteins or other AAA proteins in Fungi DB.
