# Supplementary material for: Only One of Three Bcs1 Homologs in Aspergillus fumigatus Confers Respiratory Growth
Source: J Fungi (Basel). 2023 Nov 2;9(11):1074. doi: 10.3390/jof9111074 (PMC10672213; doi:10.3390/jof9111074)
Supplement: Supplementary file 1 [file jof-09-01074-s001.zip › jof-2673429-supplementary.pdf]

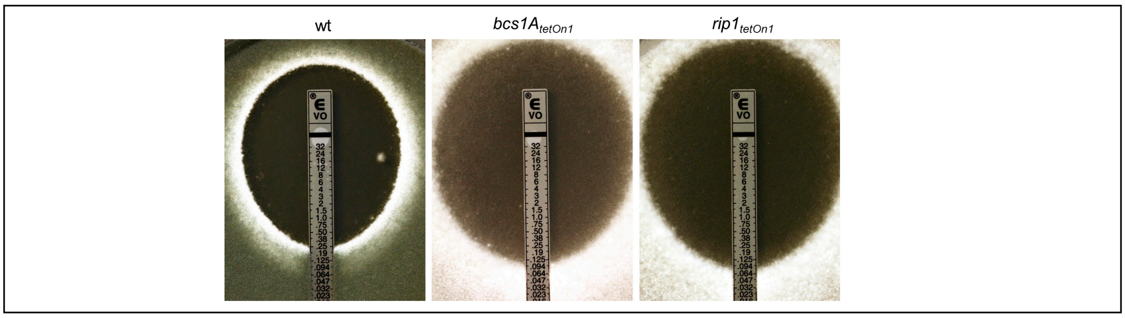

Figure S1. Reduced expression of *bcs1A* and *rip1* results in a comparable change in azole susceptibility. 1 × 10^6 conidia of the indicated strains were spread on Sabouraud agar plates. Etest strips were applied, and representative images were taken after 48 h incubation at 37 °C.

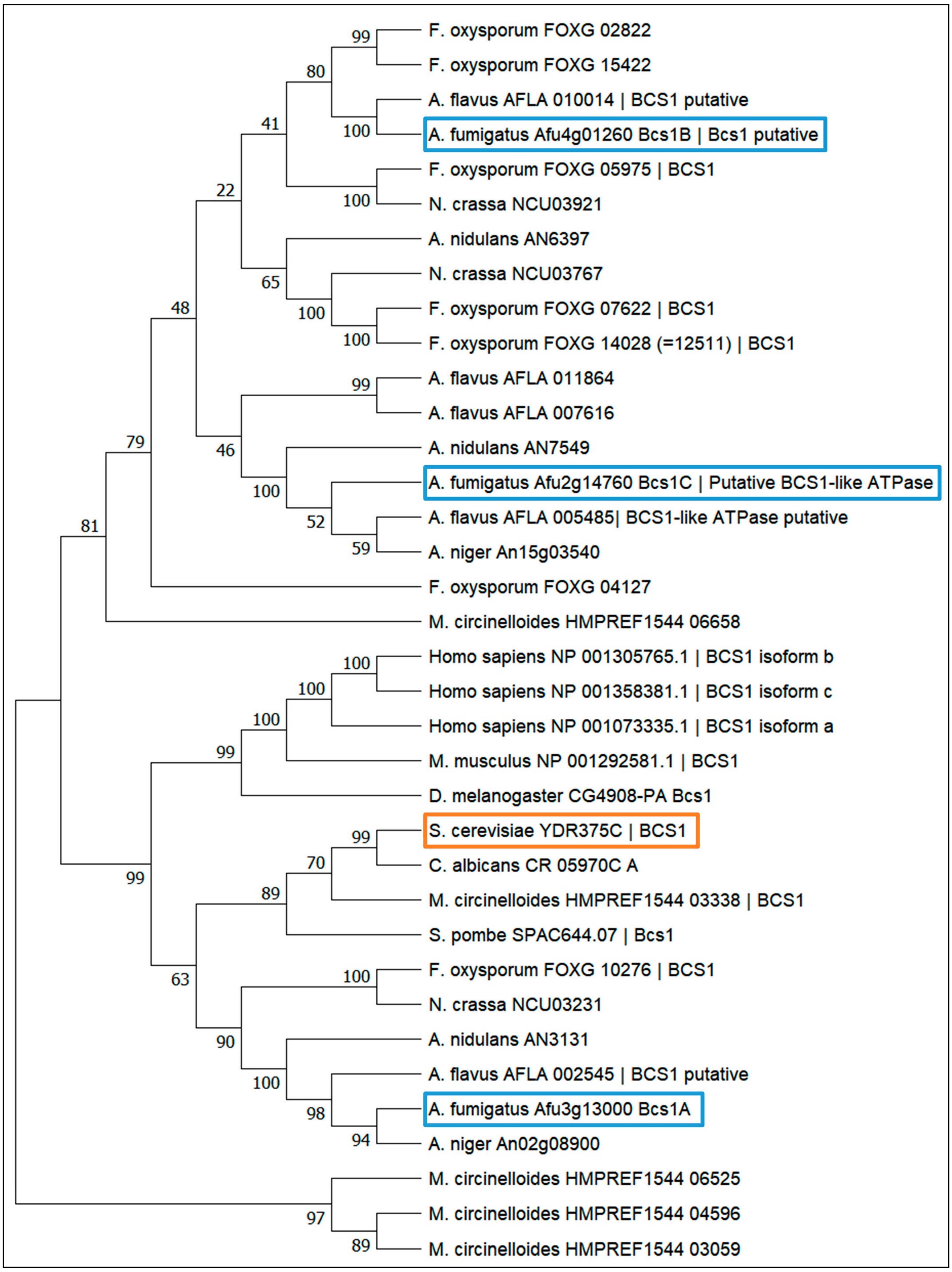

Figure S2. A neighbor-joining tree of the alignment of *S. cerevisiae*'s Bcs1 and its homologues within the indicated species was created using MEGA11. Firstly, the protein sequences were aligned using the ClustalW algorithm, then a neighbor-joining tree was generated. The values above the branches indicate the percentage of replicate trees in which the associated taxa clustered together in the bootstrap test (500 replicates). Homologues that are annotated as Bcs1 or putative Bcs1 display this information next to the respective identifier. All other homologs were annotated as hypothetical proteins or other AAA proteins in Fungi DB.
